# Utilization of Augmented Reality Head-Mounted Display for the Surgical Management of Thoracolumbar Spinal Trauma

**DOI:** 10.3390/medicina60020281

**Published:** 2024-02-06

**Authors:** Michael Ryan Kann, Miguel A. Ruiz-Cardozo, Samuel Brehm, Tim Bui, Karan Joseph, Karma Barot, Gabriel Trevino, Abigail Carey-Ewend, Som P. Singh, Matthew De La Paz, Ahmed Hanafy, Michael Olufawo, Rujvee P. Patel, Alexander T. Yahanda, Alexander Perdomo-Pantoja, Julio J. Jauregui, Magalie Cadieux, Brenton Pennicooke, Camilo A. Molina

**Affiliations:** 1 Department of Neurological Surgery, Washington University School of Medicine, St. Louis, MO 63110, USA; 2 University of Pittsburgh School of Medicine, Pittsburgh, PA 15213, USA; 3 Department of Orthopedic Surgery, University of Maryland Medical System, Baltimore, MD 21201, USA; 4 Department of Orthopedic Surgery, Washington University School of Medicine, St. Louis, MO 63110, USA

**Keywords:** augmented reality, image-guided surgery, robotics, spine surgery, spine trauma, trauma

## Abstract

*Background and Objectives*: Augmented reality head-mounted display (AR-HMD) is a novel technology that provides surgeons with a real-time CT-guided 3-dimensional recapitulation of a patient’s spinal anatomy. In this case series, we explore the use of AR-HMD alongside more traditional robotic assistance in surgical spine trauma cases to determine their effect on operative costs and perioperative outcomes. *Materials and Methods*: We retrospectively reviewed trauma patients who underwent pedicle screw placement surgery guided by AR-HMD or robotic-assisted platforms at an academic tertiary care center between 1 January 2021 and 31 December 2022. Outcome distributions were compared using the Mann–Whitney U test. *Results*: The AR cohort (n = 9) had a mean age of 66 years, BMI of 29.4 kg/m^2^, Charlson Comorbidity Index (CCI) of 4.1, and Surgical Invasiveness Index (SII) of 8.8. In total, 77 pedicle screws were placed in this cohort. Intra-operatively, there was a mean blood loss of 378 mL, 0.78 units transfused, 398 min spent in the operating room, and a 20-day LOS. The robotic cohort (n = 13) had a mean age of 56 years, BMI of 27.1 kg/m^2^, CCI of 3.8, and SII of 14.2. In total, 128 pedicle screws were placed in this cohort. Intra-operatively, there was a mean blood loss of 432 mL, 0.46 units transfused units used, 331 min spent in the operating room, and a 10.4-day LOS. No significant difference was found between the two cohorts in any outcome metrics. *Conclusions*: Although the need to address urgent spinal conditions poses a significant challenge to the implementation of innovative technologies in spine surgery, this study represents an initial effort to show that AR-HMD can yield comparable outcomes to traditional robotic surgical techniques. Moreover, it highlights the potential for AR-HMD to be readily integrated into Level 1 trauma centers without requiring extensive modifications or adjustments.

## 1. Introduction

Pedicle screw instrumentation is a surgical technique that was first introduced in 1985 for fracture stabilization in trauma settings [1,2]. It has since become a popular method used for various surgical indications of degenerative lumbar pathology due to its ability to improve postoperative recovery, minimize soft-tissue trauma, and lead to better deformity correction [2,3]. The increased use of this technique over the past decades has led to drastic technological advancements in its application. Pedicle screw placement was traditionally performed utilizing the free-hand technique, in which anatomical landmarks were used to identify the entry point and trajectory of the screw implant. This method was enhanced with intra-operative fluoroscopic and computerized tomography (CT) imaging to provide further precision to surgeons implanting pedicle screws. Nevertheless, these enhancements still posed challenges such as the shifting focus of the surgeon between an external screen and the surgical field, which decreased the workflow efficiency [4,5]. As malpositioned screws can compromise spinal stability and cause substantial morbidity due to neurologic, dural, vascular, and visceral injury [6,7,8], there is a need for a surgical method that produces highly accurate, reproducible, and precise screw placement. Robotic-assisted surgery has recently become a popular method to address this need and produces highly accurate screw placement, ranging from 94.5% to 99% [9,10]. Nevertheless, problems with the robotic-assisted technique still exist, including its lack of tactile feedback, the inability to make adjustments during screw placement, and its high acquisition costs [11,12,13,14].

The issues with currently available technologies can be magnified in trauma cases where expeditious injury assessment and the development of an accurate plan of action is of the upmost important to successful intervention. Particularly in spine surgery, the physician response has a particularly large impact on operative outcomes, and significant neurologic sequelae is possible if spinal decompression, stabilization, and reduction, when indicated, are not properly achieved. As a result, the integration of novel technological advancements into trauma medicine has the capability to drastically improve the speed and accuracy of a physician’s care response. Augmented reality head-mounted display (AR-HMD) is a novel technology that provides surgeons with a CT-guided 3-dimensional recapitulation of an individual patient’s spinal anatomy in real time [15]. In non-emergent pedicle screw placement surgeries, AR-HMD has already been shown to mitigate the limitations of other pedicle screw instrumentation techniques while producing the same level of clinical accuracy [16]. However, the use of this technology specifically in spine trauma cases has not yet been explored.

Due to the novelty of this technology and nearly no studies published in the trauma literature, the purpose of this study is to describe the use of AR-HMD in surgical thoracolumbar spine trauma. With this, we hope to determine whether the integration of AR-HMD technology into the trauma response setting affects perioperative and patient outcomes such as the surgery time, blood loss, length of stay, and postoperative pain. These results are described alongside those of a similar cohort of trauma patients who underwent robotic-assisted pedicle screw insertion, providing a comparison between the two methodologies.

## 2. Materials and Methods

### 2.1. Data Collection and Analysis

After the Institutional Review Board (IRB) approved the waiver of consent for this single-institution retrospective study (IRB 202006008), we reviewed the data obtained in all patients who underwent consecutive pedicle screw placement performed at a single academic tertiary care center between 1 January 2021 and 31 December 2022. Inclusion criteria included age older than 18 years, trauma stated as the indication for surgery designated by the International Classification of Diseases (ICD)10 code [17] (including oncologic pathologic fractures (PF)), and surgery guided by AR-HMD or robotic-assisted platforms. The demographic factors collected included sex, age, and body mass index (BMI). The specific clinical details included Charlson Comorbidity Index (CCI), surgical invasiveness index (SII), and postoperative 3-month, 6-month, and 12-month Oswestry Disability Index (ODI) scores. In addition, we collected specific surgical and instrumentation information such as the injury classification, spinal region, and number of screws placed. The analyzed perioperative outcomes included blood lost during surgery, the number of transfusion units used during surgery, minutes spent in the operating room, days spent in the hospital, the discharge disposition, and confirmation of harmonious screw placement (document intraosseous placement and screws parallel to the superior endplates, and not extending the anterior border of the vertebral body) in bilateral X-rays and complications/reoperations reported after surgery. All graphs were created using GraphPad Prism software version 9.4.1 for Mac (GraphPad Software, San Diego, CA, USA), and all tables were produced using open-source programming language R (R Foundation for Statistical Computing, Vienna, Austria) and R Studio (RStudio PBC, Boston, MA, USA) and modified in Microsoft Word (Microsoft Office, Redmond, WA, USA). The perioperative and patient-reported outcome measures (PROMs) between patients who underwent surgery guided by AR-HMD or robotic-assisted platforms were compared in SPSS using a Mann–Whitney U Test. Cost data were pulled from the Barnes Jewish standardized cost data warehouse and grouped by the operating room functional area for dissemination purposes. 

### 2.2. Surgical Technique for AR-HMD

**Figure 1:**
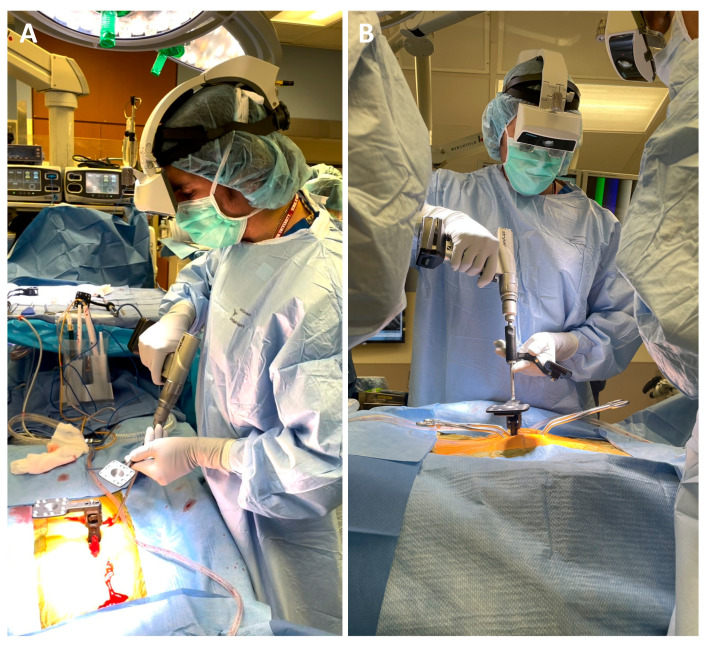
(**A**) The augmented reality head-mounted display (AR-HMD) (xvision; Augmedics, Chicago, IL, USA) unit in use including an (**B**) integrated tracking camera and translucent retina display.

**Figure 2:**
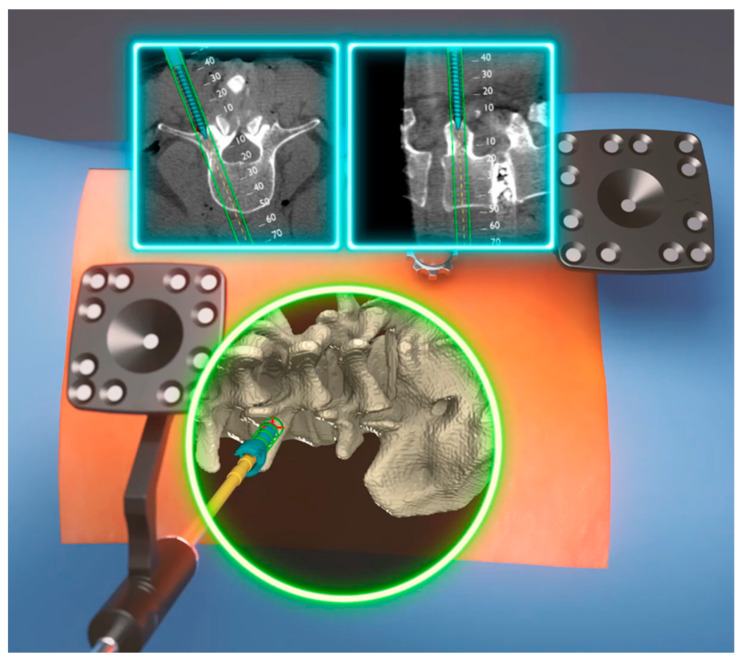
Rendered intra-headset displays images that combine 2D and 3D segments of the bone structure in the spine of sawbones. The 2D views are accurately aligned with the actual sawbones’ orientation, position, and scale. This rendered image is shown because acquiring such visualizations during an operation is not safe (Courtesy from Augmedics).

Patients were placed on a Jackson table in the prone position with intraoperative neuromonitoring and were then prepped and draped in standard sterile fashion. A registration clamp and marker were placed on a spinous process, while intraoperative 3D imaging and registration was acquired with an O-arm (Medtronic, Minneapolis, MN, USA). The level of the registration clamp and marker was chosen based on the exposure and number of planned instrumentation levels. The registration marker on the clamp was then exchanged for a tracking marker that was placed on the contralateral working side. The surgeons were fitted with an AR-HMD (xvision, Augmedics, Chicago, IL, USA) that provided a 3D overlay of the patient’s anatomy in a 1:1 size ratio alongside 2D axial and parasagittal tool trajectory projections (Figure 1 and Figure 2). 

The pedicle screws were placed percutaneously by utilizing AR computer navigation assistance, with the screw entry point selected based on anatomical landmarks and confirmed with AR computer navigation. A properly calibrated AR-tracked pedicle probe and tap were used for the cannulation of the pedicles, and a calibrated AR-tracked power screwdriver was used to drive the screw into its final position or AR-HMD assistance was used to place the screws manually. After proper screw placement, the registration clamp and patient markers were removed, and the rest of the surgery was completed utilizing standard techniques. The screw placement accuracy was confirmed intraoperatively with an O-arm or postoperatively with CT. The detailed technical workflow of the use of AR-HMD technology in spine surgery and pedicle screw insertion as well as AR-HMD visualization has been previously described [16,18,19].

## 3. Results

### 3.1. AR-HMD-Assisted Patient Cohort

A total of nine patients underwent AR-assisted percutaneous pedicle screw placement (Table 1 and Table 2). In these nine patients, 77 percutaneous pedicle screws were placed, with the mean number of screws per patient being 8.55 (range 2–16). Of these 77 screws, 39 (51%) were placed in the thoracic spine, 30 (39%) were placed in the lumbar spine, and 8 (10%) were placed in the sacral spine. Pedicle screws were placed bilaterally except for one unilaterally placed screw at T11 in patient 9.

**Figure 3:**
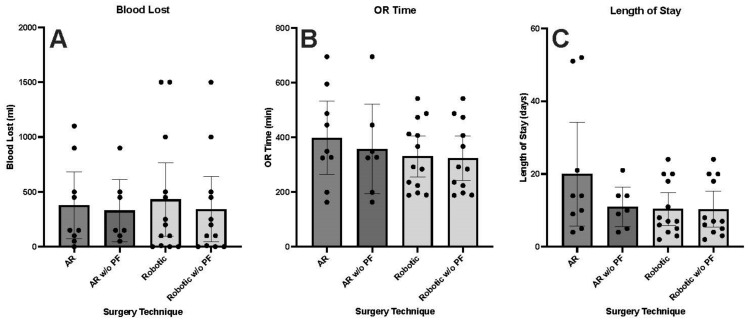

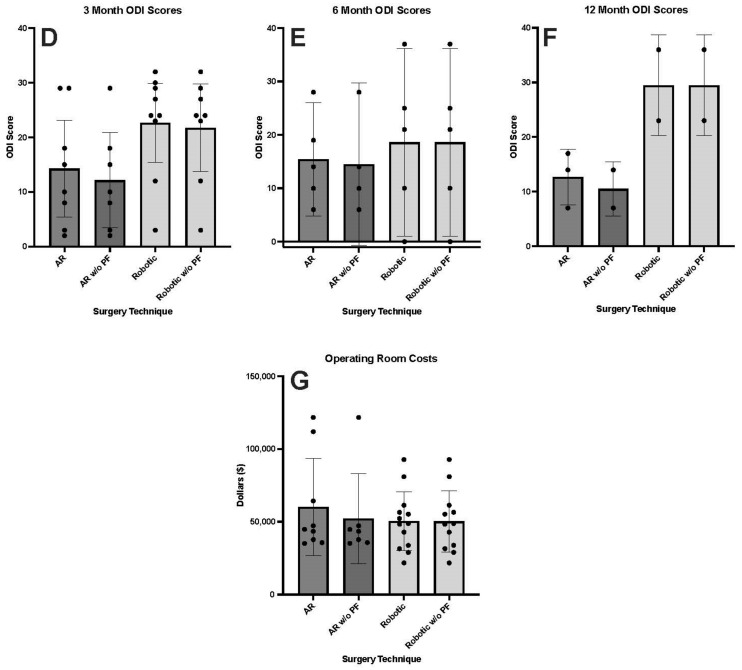
Column graphs representing the mean (**A**) milliliters of blood lost during surgery, (**B**) minutes spent in the operating room during surgery, (**C**) number of days patients were in the hospital, (**D**) Oswestry Disability Index scores three months after surgery, (**E**) Oswestry Disability Index scores six months after surgery, (**F**) Oswestry Disability Index scores twelve months after surgery, and (**G**) operating room costs for trauma patients who underwent AR-HMD or robotic-assisted pedicle screw placement surgery. This is represented for the total AR-HMD and robotic cohorts as well as the AR-HMD and robotic cohorts without those whose mechanism of injury was pathologic fracture. Error bars represent 95% confidence interval. Abbreviations: AR—augmented reality cohort, AR w/o PF—augmented reality cohort without those who suffered pathologic fractures, Robotic w/o PF—Robotic cohort without those who suffered pathologic fractures.

This cohort included four females and five males with a mean age of 66.00 years (range 57–81), a body mass index of 29.37 kg/m^2^ (range 20.47–38.90), a CCI of 4.11 (range 1–9), and a SII of 8.78 (range 4–13). There were eight white patients and one black patient. Injuries were sub-classified into one-level fracture (6), two-level fracture (2), and three-level fracture (1). The mechanism of injury included falls (4), neoplasms (2), ankylosing spondylosis (1), hardware failure (1), and motor vehicle accident (1) (Table 2). Intra-operatively, there was an average of 377.78 mL of blood lost (range 0–1100), 0.78 units of packed red blood cells (PRBCs) transfused (range 0–2), 398.33 min spent in the operating room (range 325–695), and 20 days LOS in the hospital (range 4–52) across the whole cohort. When the two patients with pathologic fractures were excluded from analysis, there was an average of 323.57 mL of blood lost (range 50–900), 357.57 min spent in the operating room (range 325–695), and 11 days LOS in the hospital (range 4–21) (Figure 3A–C). The average 3-month, 6-month, and 12-month ODI scores were 14.25 (n = 8, range 2–29), 15.40 (n = 5, range 10–28), and 12.67 (n = 3, range 7–17), respectively (Figure 3D–F). Four patients were discharged home, one patient was discharged to an intermediate care facility, two patients were discharged to skilled nursing facilities, and two patients were discharged to other rehabilitation facilities. The average operative costs associated with surgery were USD 60,218 (range $35,172–$121,647) in the full cohort and USD 52,255 (range USD 35,172–121,647) when the two patients with pathologic fractures were excluded (Figure 3G).

### 3.2. Robotic-Assisted Patient Cohort

A total of thirteen patients underwent robotic-assisted pedicle screw placement (Table 1 and Table 3). In these thirteen patients, 128 percutaneous pedicle screws were placed, with the mean number of screws per patient being 9.85 (range 4–17). Of these 123 screws, 75 (59%) were placed in the thoracic spine, 47 (37%) were placed in the lumbar spine, 2 (2%) were placed in the sacral spine, 2 (2%) were placed in the iliac spine, and 2 (2%) were placed in the pelvis. Pedicle screws were placed bilaterally, except for a unilaterally placed screw at L5 in patient 4 and T12 in patient 12.

The cohort included five females and eight males with a mean age of 55.61 years (range 23–85), a body mass index of 27.12 kg/m^2^ (range 17.57–33.91), a CCI of 3.77 (range 0–12), and an SII of 14.23 (range 6–33). All patients were white. Injures were categorized into one-level fracture (10), two-level fracture (2), and eleven-level fracture (1). The mechanism of injury included falls (6), neoplasms (1), motor vehicle accidents (5), and a falling object (1) (Table 3). Intra-operatively, there was an average of 431.54 mL of blood lost (range 0–1500), 0.46 units of PRBC transfused (range 0–3), 330.61 min spent in the operating room (range 189–542), and 10.38 days LOS in the hospital (range 2–24) across the whole cohort. When the one patient with a pathologic fracture was excluded from analysis, there was an average of 342.50 mL of blood lost (range 0–1500), 323.83 min spent in the operating room (range 189–542), and 10.33 days LOS in the hospital (range 2–24) (Figure 3A–C). The average 3-month, 6-month, and 12-month ODI scores were 22.67 (n = 9, range 3–37), 18.60 (n = 5, range 0–37), and 29.50 (n = 2, range 23–36), respectively (Figure 3D–F). Nine patients were discharged home, one patient was discharged to home health care, two patients were discharged to other rehabilitation facilities, and one patient was discharged to a skilled nursing facility. The average operative costs associated with the surgery were USD 50,397 (range USD 21,799–92,719) in the full cohort and USD 50,240 (range USD 21,799–$92,719) when the one patient with a pathologic fracture was excluded (Figure 3G). 

### 3.3. Comparison between AR-HMD and Robotic-Assisted Patient Cohorts

No significant difference was found in any of the outcome metrics between trauma patients who underwent AR-assisted pedicle screw placement and trauma patients who underwent robotic-assisted pedicle screw placement (Figure 3). This held true both when patients with pathologic fractures were included and when patients with pathologic fractures were excluded from analysis. Despite the observed trend of lower 12-month ODI scores in the AR-HMD-assisted patient cohort compared to the robotic-assisted patient cohort, this difference was not statistically significant (*p* = 0.083). The only significant difference found between the two patient cohorts of all recorded metrics was in the SII score (*p* = 0.036).

## 4. Clinical Case Examples

### 4.1. AR-HMD

A 62-year-old female with a medical history notable for cognitive delay came to the clinic. The medical history obtained from both the patient as well as her assisted living facility and primary caretaker revealed that she has been experiencing a progressive decrease in function alongside severe weight loss. She had lost approximately 20 lbs and had been refusing to get out of bed. When she did, she resorted to crawling or scooting around the floor in order to get around. While initially believed to be a behavioral issue, further workup and CT revealed that she had a comminuted complex fracture of the S1 vertebral body with extension to the bilateral sacral ala line S1 foramen. This had the potential appearance of a pathologic fracture and an MRI demonstrated the presence of an infiltrated lytic lesion of the S1 and S2 vertebral body with extension into the sacral spinal canal and out the sacral spinal foramen. A preoperative angiogram showed no significant hypervascularity and a preoperative biopsy was inconclusive. Given the lack of diagnosis and the need to decompress the spinal canal as well as to restore the mechanical integrity of her spine, a minimally invasive L4 to pelvis arthrodesis and an S1 and S2 laminectomy were performed, as well as tumor debulking for the decompression of the spinal canal and to obtain tissue for further diagnosis. The AR-HMD system was used during bilateral pedicle screw fixation at L4 and L5 as well as for bilateral sacroiliac fixation, with dual screws on both the left and the right side crossing the sacroiliac junction from the S2 vertebral body and S3 vertebral body level into the iliac wings. The instrumentation placement was checked using an intraoperative CT scan. After successful screw placement was confirmed, rods were passed percutaneously and bilaterally across L4-5 into the midline exposure and side connected to two separate rods affixed to the S2 and S3 sacroiliac screws bilaterally. The placement of the hardware and rods was confirmed with a lateral portable X-ray. Following surgery, the patient was discharged. The surgery was performed from 10:00 a.m. to 7:55 p.m. and during the 595 min surgery, the patient lost minimal blood and required one transfusion unit. She was in the hospital for a total of 52 days due to other non-operative complications before being released home. Post-operatively, the patient reported ODI scores of 15, 10, and 14 at 3 months, 6 months, and 12 months after surgery, respectively.

### 4.2. Robotic

A 63-year-old female with a medical history significant for morbid obesity, hypertension, chronic pain, obstructive sleep apnea, and depression presented to an outside hospital after falling down 14 stairs. She reported hitting her head during the fall but denied any loss of consciousness or neck pain. She complained of severe low back pain but denied any numbness, tingling, or loss of bowel or bladder function. Her physical exam was limited by pain but concerning for proximal lower extremity weakness. CT of the lumbar spine revealed evidence of an acute-appearing L2 burst fracture with retropulsion and moderate to severe spinal stenosis. Further imaging showed that the patient had significant premorbid sagittal and coronal plane deformity. The management of her acute fracture and posttraumatic stenosis was prioritized, and surgery for her deformity was deferred to later if necessary. To this end, an L1–L3 posterior decompression with T12–L4 posterior spinal fusion was planned with robotic assistance. The intraoperative spine robot was used to register the patient’s spine to the preoperative CT scan, and the navigated guide was used to cannulate the pedicles of T12, L1, L3, and L4 for bilateral pedicle screw placement. Rods were then cut to length, contoured to shape, and affixed to screws using a torque/counter-torque device. The implant placement was evaluated with AP and lateral fluoroscopy, followed by posterior decompression. During the 445 min procedure, the patient lost about 1500 cc of blood and required two units of packed RBC transfusion. Following surgery, the patient remained in the hospital for 6 days and was discharged due to postoperative pain control and oxygen requirements likely related to the patient’s pre-existing co-morbidities. The patient reported ODI scores of 24, 21, 23 at 3-month, 6-month and 12-month intervals after surgery, respectively.

## 5. Discussion

AR-HMD represents an innovative FDA-approved stereotactic navigation platform designed to address numerous challenges inherent in manual computer-navigated and robotic-assisted surgical technologies. The system takes advantage of an integrated tracking camera and translucent retina display mounted directly onto the headgear to project a 3D and 2D navigational virtual interface directly over the top of the surgical field in a way that anatomically matches the spine orientation, size, and location. This enables surgeons to maintain unwavering concentration on the surgical field, eliminating the attentional shifts that are prevalent in both manual and robotic-assisted navigation systems. This clinical series is the first to our knowledge to report on the perioperative and patient-reported outcomes of AR-HMD-guided pedicle screw placement surgery for thoracolumbar trauma at a single tertiary academic institution. We reported on the blood lost during surgery, the transfusion units used, the minutes spent in the operating room, the length of stay in the hospital, the ODI scores at 3, 6, and 12 months post-surgery, and operative room cost. We reported on these same outcomes in a cohort of thoracolumbar trauma patients who underwent robotic-assisted pedicle screw placement surgery. Our findings revealed similar outcomes between thoracolumbar trauma patients who underwent robotic-assisted or AR-HMD-assisted pedicle screw placement surgery. As a result, this case series contributes to the existing literature supporting the use of AR-HMD technology in pedicle screw placement surgery, specifically in its adoption in the setting of the instrumentation of the spine during acute trauma.

Previous studies have been published on the clinical accuracy of AR-HMD-assisted pedicle screw placement surgery [16,20,21] and robotic-assisted pedicle screw placement surgery, graded using the Gertzbein–Robbins scale. The use of the AR-HMD system on cadavers has previously been shown to result in a 94.6% and 99.1% accuracy grade on the Gertzbein–Robbins scale for lumbosacral and thoracic pedicle screw placement [15,22]. When used in patients, AR-HMD technology has been shown to result in 100% accuracy for 6 screws placed in a single patient [19], 98% accuracy for 205 screws placed in 28 patients across a variety of surgical indications [16], and 97.1% accuracy for 218 screws in 32 patients undergoing thoracolumbar fusion [23]. This accuracy was duplicated for percutaneous thoracolumbar pedicle screw placement, with 100% accuracy across 63 screws in nine patients [18]. It was also shown to result in excellent clinical accuracy for pedicle screw placement during a posterior en bloc L1 corpectomy for a chordoma [24]. Additionally, the precision of pedicle screw placement has been shown to be improved with AR-HMD technology [25]. In comparison, robotic-assisted surgery performed using the SpineAssist robot achieved a Gertzbein–Robbins-graded clinical accuracy of 97.9% for 487 screws in 112 patients, which later improved to 98.5% [26] and 98.7% [27] accuracy with modifications made to the SpineAssist platform. Other robotic-assisted platforms, including ExcelsiusGPS, ROSA, and TianJi, have reported a clinical accuracy of between 96.6% and 100% across multiple studies [20,27,28,29,30,31,32]. As a result, the existing literature supports a similar or improved clinical accuracy for pedicle screw placement when utilizing AR-HMD technology compared to robotic-assisted platforms.

While the clinical accuracy of AR-HMD-assisted spine surgery has shown to be similar to that achieved in robotic-assisted spine surgery, none of the previous studies have reported on the differences in perioperative and patient-reported outcomes between the two surgical techniques. Perioperative metrics are important measures of hospital efficiency, patient care, and the effective use of hospital resources. This series revealed similar perioperative and patient reported outcomes in patients who underwent robotic-assisted or AR-HMD-assisted pedicle screw placement surgery with regard to the blood lost during surgery, transfusion units used, operating time, LOS, and 3-, 6-, and 12-month ODI scores. While not statistically different, it is important to note that the average LOS for the AR-assisted patient cohort was considerably longer than that for the robotic-assisted patient cohort. Nevertheless, this was primarily due to patients whose mechanism of injury was an oncologic pathologic fracture and who, as a result, had increased LOS caused by complications not related to the use of AR technology. When excluding patients with oncologic pathologic fractures from both the AR-HMD and robotic-assisted cohorts to control for this patient heterogeneity, the disparity in LOS between the two groups drastically diminishes to 11 days in the AR-HMD group and 10.33 days in the robotic-assisted cohort.

Furthermore, a comparative evaluation of the operative room costs has large implications for the cost-saving potential of novel technology. While our case series revealed no statistical difference in the operative costs between AR and robotic-assisted technologies in spine surgery, the surgical operative costs for the AR-assisted patient cohort were considerably larger than the robotic-assisted patient cohort. Again, this is primarily due to patients whose mechanism of injury was driven by an oncologic pathologic fracture and who, as a result, had additional complications that needed to be addressed during surgery. This could include tumor removal as well as the greater use of hemostatic products and biologics that drastically increased the surgical operative costs but were not related to the use of AR technology for pedicle screw placement. When excluding patients with oncologic pathologic fractures from both the AR-HMD and robotic-assisted cohorts to control for this patient heterogeneity, the disparity in operative costs between the two groups drastically diminishes from a difference of USD 9821 to a difference of only USD 2015. Therefore, our findings reveal similar operating room costs associated with AR-assisted or robotic-assisted spine surgery for trauma indications. As AR technology requires lower upfront costs, this could help a hospital/healthcare system invest in this technology, with potentially improved long-term returns.

Lastly, patient-reported outcomes, such as ODI scores, can act as indicators for the long-term interventional utility of spine surgery, which greatly impacts patients’ quality of life. The patient response rate needed to evaluate long-term changes in ODI scores was limited within our case series, and as AR technology continues to become more widely used in spine surgery, further investigation is necessary in a larger patient cohort comparing the patient-reported outcomes and interventional utility between AR and robotic-assisted spine surgery techniques.

While previous studies investigating the use of AR-HMD technology in spine surgery include patients with a variety of surgical indications [33], our case series focused particularly on trauma patients. In trauma cases, physicians must act quickly and accurately to diagnose, treat, and surgically intervene in patients who present with significant spine injury. With this response having a large impact on patients’ surgical outcomes, innovations in the field that can improve the accuracy and speed of the physician response to trauma are of great importance. Our study suggests that AR technology has the ability to produce similar operative outcomes to robotic-assisted technologies in spine trauma cases. As this technology is still relatively new to the field, one of the primary challenges for its integration into level 1 trauma centers is the limited number of experienced surgeons who are proficient in its usage. While previous studies focused on surgeon feedback have found the AR-HMD system to be relatively intuitive and user friendly, there remains a learning curve associated with its adoption [15]. The AR-HMD headset may cause a disorienting or discomforting experience for unfamiliar surgeons encountering the new virtual interface on top of the surgical field. However, these initial hurdles can be readily surmounted through the ongoing utilization of and accumulation of experience with this innovative technology integrated into the surgical workflow. Future studies in a larger patient cohort are needed to compare the impact that AR and robotic technologies have on the clinical workflow, workforce efficiency, and the speed and accuracy of a physician’s response in the context of spine surgical trauma.

### Limitations

The primary limitation of this study is its low number of trauma patients (n = 22) and pedicle screws (n = 205) for both AR-HMD and robotic-assisted pedicle screw placement surgery. However, as new technologies arise, it is important to evaluate these early outcomes as an early proof of concept study to determine the safety and efficacy of this technology. Studies investigating the perioperative and patient-reported outcomes in a greater number of patients with trauma indications for surgery are necessary to determine the full utility of AR-HMD technology in spine trauma. Additionally, the AR-HMD-guided surgeries were performed at one institution by one surgeon who is familiar with AR-HMD technology. Therefore, our results cannot be generalized across different institutions or across various surgeons who have a different level of experience and expertise with AR-HMD technology. Nevertheless, this case series represents the first study performed in order to explicitly investigate the outcomes associated with AR-HMD technology in spine trauma cases for pedicle screw placement and paves the way for a future exploration of the use of this technology across other surgical indications. This study does not report accuracy using GRS scores as the post-operative CT scans that are needed for the calculation of the GRS grades are not routinely performed in all patients and are reserved for specific indications. We acknowledge the importance of accuracy in our outcomes, and in response, we included confirmation of harmonious screw placement on bilateral radiographic projections.

As we are studying trauma patients, we were not able to obtain pre-operative ODI scores from our patient cohort and were limited to ODI scores obtained at 3-month, 6-month, and 12-month intervals after surgery. As a result of not being able to calculate the pre- and post-operative change in ODI scores, we cannot draw any conclusions based on our data regarding the effectiveness of AR-HMD or robotic-assisted surgery at decreasing disability caused by lower back pain in trauma cases. Likewise, there was a consistent decrease in the number of ODI responses recorded over the 3-month, 6-month, and 12-month intervals, from eight to five to three responses in AR cases and nine to five to two responses in robotic-assisted cases, respectively. This was either due to a lack of patient follow-up or the patient not properly being administered the ODI survey at follow-up appointments. This could introduce attrition bias into our ODI scores, as patients either with higher or lower levels of disability for lower back pain may be more likely to schedule and attend follow-up appointments after spine surgery.

## 6. Conclusions

This case series demonstrates the potential utility of AR-HMD in trauma patients requiring spinal surgical intervention. The mean blood loss, length of hospital stay, transfusion units, and ODI outcomes were comparable to the outcomes observed in robotic-assisted surgery. While the urgency to treat a spinal pathology constitutes a major hurdle to the adoption of novel technologies in spine surgery, this study is an early attempt to demonstrate that AR-HMD can provide adequate outcomes that are similar to commonly used robotic surgical practices and can be quickly adopted in Level 1 trauma centers without extensive adaptations. The future validation of this case series with a larger patient cohort is necessary to fully determine the utility of AR-HMD in spine trauma cases.

## Figures and Tables

**Table 1:** Patient demographic and clinical information.

	Value	
Variable	AR Assisted	Robotic Assisted
Female sex	4 (44%)	5 (38%)
White race	8 (89%)	13 (100%)
Mean age, yrs	66.00 ± 10.33	55.62 ± 19.75
Mean BMI, kg/m^2^	29.37 ± 6.09	27.12 ± 5.12
Charlson Comorbidity Index	4.11 ± 2.98	3.77 ± 4.21
Surgical Invasiveness Index	8.78 ± 3.19	14.23 ± 7.08 *
Injury Classification		
One-level fracture	6 (67%)	10 (77%)
Two-level fracture	2 (22%)	2 (15%)
Three-level fracture	1 (11%)	0 (0%)
Eleven-level fracture	0 (0%)	1 (8%)
Mechanism of Injury		
Falls	4 (44%)	6 (46%)
Neoplasms	2 (22%)	1 (8%)
Ankylosing spondylosis	1 (11%)	0 (0%)
Hardware failure	1 (11%)	0 (0%)
Motor Vehicle Accident	1 (11%)	5 (38%)
Falling object	0 (0%)	1 (8%)
Total No. of Screws	77	128
Thoracic	39 (51%)	75 (59%)
Lumbar	30 (39%)	47 (37%)
Sacral	8 (10%)	2 (2%)
Iliac	0 (%)	2 (2%)
Pelvis	0 (%)	2 (2%)
Discharge Disposition		
Home	4 (44%)	9 (69%)
Rehabilitation facility	2 (22%)	2 (15%)
Home health care	0 (0%)	1 (8%)
Intermediate care facility	1 (11%)	0 (0%)
Skilled nursing facility	2 (22%)	1 (8%)

Values represent the number of patients (%) or mean ± SD. * Statistically significant difference between AR and Robotic-assisted patient cohorts (*p* = 0.036).

**Table 2:** Spinal surgical procedures for each AR-assisted procedure in trauma settings, including the number and location of percutaneous pedicle screws.

Patient No.	Surgical Procedure	No. and Location of Pedicle Screws Placed	Harmonious Position on Biplanar Projections	Complications/Reoperations
1	Percutaneous posterior spinal instrumentation from L4 to L5; open sacroiliac fixation with dual screws at the S2–S3 vertebral body level.	4 lumbar, 2 sacral	Yes	Lumbar pseudoarthrosis ^#^
2	Percutaneous posterior spinal instrumentation from T7 to L2; T10 and T11 laminectomy; bilateral facetectomies at T10–11 and T11–T12; bilateral T11 pediculectomies; removal of posterolateral and ventral tumor.	12 thoracic, 4 lumbar	Yes	None
3	Spinal arthrodesis from L2 to S1; posterior spinal instrumentation from L2 to S1; pelvic fixation using an S2 alar iliac screw technique; posterior column osteotomies and sublaminar decompression at L2–L3, at previously arthrodesis L3–L4, and L5–S1.	8 lumbar, 4 sacral	Yes	None
4	Percutaneous posterior spinal instrumentation from T9 to L2; posterior spinal arthrodesis from T10–T11; complete laminectomies including foraminotomies at T10 to T11.	8 thoracic, 4 lumbar	Yes	None
5	Posterolateral and midline arthrodesis from L4 to S1; pelvic fixation using S2 alar iliac screw technique.	2 sacral	Yes	None
6	Percutaneous posterior spinal instrumentation from T12 to L4; laminectomy of the L2 vertebra for decompression.	2 thoracic, 6 lumbar	Yes	None/Elective hardware removal 12 months
7	Percutaneous posterior spinal instrumentation from T8 to T12; spinal arthrodesis from T8 to T12.	4 thoracic	Yes	None
8	Percutaneous posterior spinal instrumentation from T8 to T11.	8 thoracic	Yes	None
9	Percutaneous posterior spinal instrumentation from T10 to L2.	5 thoracic *, 4 lumbar	Yes	None

* One patient received unilateral pedicle screw placement at T11. ^#^ Underwent an L4–S1 anterior lateral interbody fusion and L3-pelvis posterior spinal fusion for correction.

**Table 3:** Spinal surgical procedures for each robotic-assisted procedure in trauma settings, including the number and location of percutaneous pedicle screws.

Patient No.	Surgical Procedure	No. and Location of Pedicle Screws Placed	Harmonious Position on Biplanar Projections	Complications/Reoperations
1	Posterior spinal instrumentation S1 to the pelvis.	2 iliac, 2 sacral	Yes	None/Elective hardware removal after 8 months
2	Laminectomies from L1 to L3 for decompression; T12–L4 posterior spinal instrumentation; spinal arthrodesis from T12 to L4	2 thoracic, 6 lumbar	Yes	None
3	Posterior spinal instrumentation from T7 to T11; spinal arthrodesis from T7 to T11.	8 thoracic	Yes	None/Elective hardware removal after 10 months
4	Posterior spinal instrumentation from L1 to the pelvis; fusion spinal–posterior lumber/thoracic with instrumentation—T8–L1.	10 thoracic, 5 lumbar *, 2 pelvis	Yes	None/Elective hardware removal 12 months
5	Laminectomies from T7–T8 for decompression; posterior spinal instrumentation from T5 to T10; spinal arthrodesis T5–T10.	8 thoracic	Yes	None
6	Laminectomies and facetectomy at the T10–T11 and T11–T12 levels; posterior spinal instrumentation from T9 to L1; spinal arthrodesis from T9–T11; corpectomy/partial vertebral column resection with anterior column reconstruction at T11.	6 thoracic, 2 lumbar	Yes	None
7	Percutaneous posterior spinal instrumentation T10 to L3; spinal arthrodesis from T10 to L3.	6 thoracic, 6 lumbar	Yes	None
8	Bilateral facetectomies at L2–L3; partial medial facetectomies L3 to L4; percutaneous posterior spinal instrumentation L1 to L5; spinal arthrodesis from L2 to L4.	8 lumbar	Yes	None
9	Percutaneous posterior spinal instrumentation from T5 to T7.	6 thoracic	Yes	None
10	Posterior spinal instrumentation from T6 to L1; spinal arthrodesis from T6 to L1.	14 thoracic, 2 lumbar	Yes	None
11	Percutaneous posterior spinal instrumentation from T10 to L2.	6 thoracic, 4 lumbar	Yes	None
12	Laminectomies and medical facetectomies from T11 to L1; right T12 transpedicular decompression for ventral spinal cord decompression; posterior instrumented fusion from T9 to L3; spinal arthrodesis from T9 to L3.	7 thoracic ^†^, 6 lumbar	Yes	None
13	Percutaneous spinal instrumentation from T12 to L4.	2 thoracic, 8 lumbar	Yes	None

* One patient received unilateral pedicle screw placement at L5. ^†^ One patient received unilateral pedicle screw placement at T12.

## Data Availability

We recognize the importance of data transparency in scientific research. However, due to the sensitive nature of the data used in this study, which includes confidential health information, it cannot be made publicly available. To ensure data integrity and responsible usage, we have established a process for data requests. Researchers interested in accessing the data can contact the corresponding author and must agree to adhere to our data usage policies.
